# Epac1 and Glycyrrhizin Both Inhibit HMGB1 Levels to Reduce Diabetes-Induced Neuronal and Vascular Damage in the Mouse Retina

**DOI:** 10.3390/jcm8060772

**Published:** 2019-05-31

**Authors:** Li Liu, Youde Jiang, Jena J. Steinle

**Affiliations:** Department of Ophthalmology, Visual and Anatomical Sciences, Wayne State University School of Medicine, Detroit, MI 48201, USA; lliu@med.wayne.edu (L.L.); youdejiang55@gmail.com (Y.J.)

**Keywords:** HMGB1, Epac1, retinal vasculature, diabetic retinopathy, permeability, inflammatory mediators, SIRT1, glycyrrhizin

## Abstract

The role of high mobility group box 1 (HMGB1) in acute diabetic retinal damage has been demonstrated. We recently reported that glycyrrhizin, a HMGB1 inhibitor, protected the diabetic retina against neuronal, vascular, and permeability changes. In this study, we wanted to investigate the role of exchange protein for cAMP 1 (Epac1) on HMGB1 and the actions of glycyrrhizin. Using endothelial cell specific knockout mice for Epac1, we made some mice diabetic using streptozotocin, and treated some with glycyrrhizin for up to 6 months. We measured permeability, neuronal, and vascular changes in the Epac1 floxed and knockout mice. We also investigated whether Epac1 and glycyrrhizin work synergistically to reduce the retinal inflammatory mediators, tumor necrosis factor alpha (TNFα) and interleukin-1-beta (IL1β), as well as sirtuin 1 (SIRT1) levels. Epac1 and glycyrrhizin reduced inflammatory mediators with synergistic actions. Glycyrrhizin also increased SIRT1 levels in the Epac1 mice. Overall, these studies demonstrate that glycyrrhizin and Epac1 can work together to protect the retina. Finally, glycyrrhizin may regulate HMGB1 through increased SIRT1 actions.

## 1. Introduction

Diabetic retinopathy remains the leading cause of vision loss in working age adults. Numerous drugs have been tested for treatment of diabetic retinopathy with some having success for proliferative disease or macular edema, yet, these also have unwanted side effects. There remains an unmet need to develop novel pathways to inhibit retinopathy or prevent progression. The role of inflammation has become increasingly important in the development and progression of diabetes as a key regulator of retinal damage [1,2,3,4,5]. Our ongoing research supports the link between inflammation and diabetic retinopathy. While inflammation is a key player in diabetic retinopathy, the upstream regulation of these inflammatory mediators remains elusive.

One potential upstream regulator of retinal inflammation is exchange protein for cAMP 1 (Epac1). Both Epac1 and Epac2 have been localized in the retina [6], are expressed by bovine and human retinal endothelial cells, and shown to play a role in leukostasis. We recently demonstrated that Epac1 is a potential key signaling protein in β-adrenergic receptor actions to protect the retina against leukostasis and inflammatory mediators [7]. In this work, data indicated that only Epac1, not Epac2, has actions on human retinal endothelial cell regulation of inflammatory mediators. Epac1 can serve as an alterative pathway for β-adrenergic receptor/cAMP activation of downstream pathways [8]. Studies have also shown that Epac1 regulates vascular endothelial cell permeability [9]. We recently reported that Epac1 is protective to the retina in an ischemia/reperfusion (I/R) model of retinal stressors [10].

In addition to traditional inflammatory cytokines, studies have also suggested that diabetes may activate danger associated molecular pattern receptors (DAMPs). One of these DAMPs is high mobility group box 1 (HMGB1). Work in a *Pseudomonas aeruginosa* keratitis model showed that glycyrrhizin, a HMGB1 inhibitor, significantly reduced HMGB1 levels and bacterial load [11]. Glycyrrhizin is a natural anti-inflammatory factor in licorice that inhibits HMGB1 activities through direct binding to HMGB1 [12]. In acute diabetic studies, glycyrrhizin reduced HMGB1, ERK1/2, caspase 3 and glutamate levels [13]. We have used glycyrrhizin to show that inhibition of HMGB1 protected the retina against I/R-induced damage [14], as well as chronic diabetes-induced damage [15].

In this study, we wanted to focus on the role of Epac1 upstream of HMGB1 in the diabetic retinal vasculature. We used diabetic Epac1 floxed and endothelial cell specific knockout KO mice alone or treated with glycyrrhizin to investigate whether Epac1 could inhibit HMGB1 to protect the diabetic retina, as well as whether Epac1 and glycyrrhizin work synergistically to protect the retinal against diabetes-induced neuronal, vascular, and permeability changes.

## 2. Experimental Section

### 2.1. Mice

Epac1 floxed mice (B6;129S2-Rapgef3^tm1Geno/J^ mice) and B6 FVB-Tg (cdh5-cre)7Mlia/J Cre mice were purchased from Jackson Laboratories. After 2 generations, Epac1 floxed mice were bred with cdh5-Cre mice to generate conditional knockout mice in which Epac1 is eliminated in vascular endothelial cells [7]. At 3 months of age, both male and female Epac1 floxed and Epac1 Cre-Lox mice were used for experiments.

We also performed Western blotting on retinal samples from male C57BL/6J mice purchased from Jackson Laboratories at 8 weeks of age. All mouse experiments were approved by the Institutional Animal Care and Use Committee at Wayne State University (Protocol# 17-07-301) and adhere to the Animal Policy of the Association for Research in Vision and Ophthalmology.

Mice were made diabetic by 60 mg/kg injections of streptozotocin dissolved in citrate buffer for up to 5 consecutive days. Control mice received citrate buffer only. Glucose measurements were done biweekly, with glucose levels >250 mg/dL were considered diabetic. Mice were not fasted before blood glucose measurements, and glucose measurements were taken on blood samples obtained via tail vein, with samples measured by a hand-held measurement device. Table 1 provides body weights and glucose measurements for all Epac1 and Epac1 Cre Lox mice. Measurements of body weights and blood glucose for the C57BL/6J mice can be found in our recent publication [15].

A subset of the control and diabetic mice were treated with glycyrrhizin in their drinking water (150 mg/kg/day) [13]. Mice were maintained on the drinking water for up to 6 months.

### 2.2. Permeability

Analyses of vascular leakage were done on control and diabetic mice alone and following glycyrrhizin treatment two separate ways. Fluorescein angiography (FA) was done on a dilated pupil using tropicamide ophthalmic solution, under ketamine and xylazine anesthesia. 150 µL of AK-FLUOR (1% W/V, Akorn Inc., Lake Forest, IL, USA) was injected intraperitoneally. Retinal vessel leakage was photographed using a Micron IV (Phoenix Research Labs, Pleasanton, CA, USA). Images were obtained less than 5 min after injection of the dye.

In addition to FA, some mice were transfused with 200 µL Evans blue (0.5% in saline, Sigma Aldrich, St. Louis, MO, USA) via the tail vein. Forty-five minutes after infusion, mice were euthanized using CO_2_ and cervical dislocation. Retinas were removed, placed into 100 µL formamide, and incubated for 48 h at 55 °C. Tubes were then centrifuged and transferred to a 96 well plate. The absorbance of the retina was measured at 610 [16].

### 2.3. Neuronal Measurements

After 2 months of diabetes or diabetes + glycyrrhizin treatment, a subset of each group of mice was sacrificed for measurements of neuronal thickness, as we have previously published exception of staining with hematoxylin and eosin instead of toluidine blue [17]. Ten micrometer sections were taken from regions throughout the retina. Analyses of retinal thickness and cell numbers for each retinal layer were assessed from the same regions in each retina, as we have done in the past [17,18].

### 2.4. Vascular

At 6 months of diabetes or treatment, all remaining mice were sacrificed. Mice were processed for measurements of capillary degeneration as we have done in the past [19,20].

### 2.5. Reactive Oxygen Species

Some samples from all groups of mice at both 2 and 6 months of diabetes and treatment were processed for measurement of reactive oxygen species using the DCFDA method. Briefly, equal protein from each group was loaded into a black 96 well plate and treated with the DCFDA in triplicate, and read on a fluorescent plate reader set with an excitation of 485 nm and emission at 530 nm. Some wells were left blank, and some wells only received the DCFDA reagent. The blanks and dye only wells were subtracted from the raw data [21]. Data are plotted as the fluorescence intensity.

### 2.6. Western Blotting

At both 2 and 6 months of diabetes and/or glycyrrhizin treatment, whole retinal lysates were collected into lysis buffer containing protease and phosphatase inhibitors. Equal amounts of protein from the cell extracts were separated on the pre-cast tris-glycine gel (Invitrogen, Carlsbad, CA, USA) and blotted onto a nitrocellulose membrane. After blocking in TBST (10 mM Tris-HCl buffer, pH 8.0, 150 mM NaCl, 0.1% Tween 20) and 5% (w/v) BSA, membranes were treated with Epac1, HMGB1, SIRT1 antibodies (Abcam, Cambridge, MA, USA), and beta actin (Santa Cruz Biotechnology, Santa Cruz, CA, USA) primary antibodies followed by incubation with horseradish labeled secondary antibodies. Antigen-antibody complexes were detected by a chemilluminescence reagent kit (Thermo Scientific, Pittsburgh, PA, USA). Data were acquired using an Azure C500 (Azure Biosystems, Dublin, CA, USA). Western blot analyses were done using Image Studio Light software.

### 2.7. ELISA

A TNFα ELISA (Fisher Scientific, Pittsburgh, PA, USA) was done according to manufacturer’s instructions, with the exception that samples were exposed to primary antibody for 24 h and 100 µg of protein was loaded into all wells. The IL-1β ELISA was performed following the manufacturer’s instructions with the exception that 120 µg protein loaded into all wells, and the primary antibody was incubated overnight.

### 2.8. Statistics

A one-way ANOVA with Student–Newman–Keuls post-hoc test was used for data analyses. Data are mean + standard error of the mean (SEM) unless stated otherwise. *p* < 0.05 was considered statistically significant. Comparisons were made between Epac1 floxed vs. CreLox, Epac1 floxed + STZ vs. Epac1 CreLox + STZ, Epac1 floxed + STZ vs. Epac1 + STZ + Gly, and Epac1 floxed + STZ + Gly vs. Epac1 CreLox + STZ + Gly to determine the effects of diabetes, HMGB1 inhibition vs. Epac1 actions and whether Epac1 and glycyrrhizin are synergistic in protecting the diabetic retina.

## 3. Results

### 3.1. Glycyrrhizin Protected against Diabetes-Induced Permeability Changes

**Figure 1:**
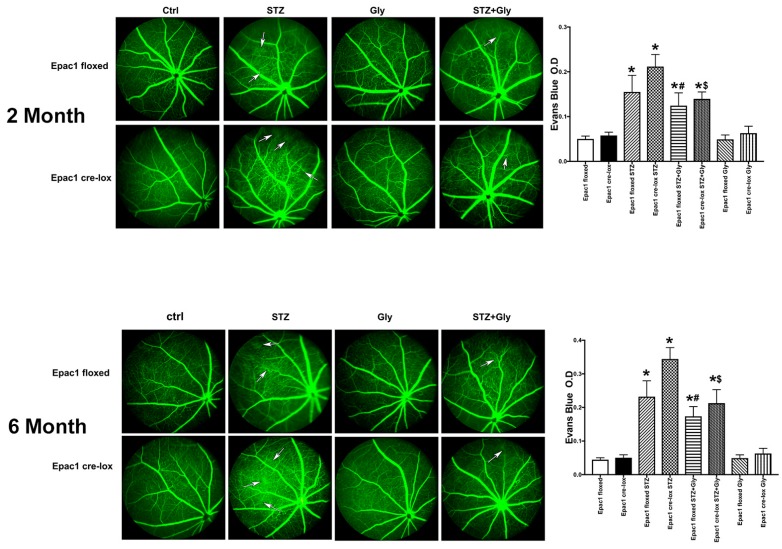
Fluorescien angiography and Evan’s blue measurement of retinal permeability. Data are from mice at 2 months (**top**) and 6 months (**bottom**) of diabetes (STZ) in Epac1 floxed and CreLox mice alone or treated with glycyrrhizin. White arrows point to locations of vascular leakage. Data are mean ± SEM * *p* < 0.05 vs. Epac1 floxed, # *p* < 0.05 is Epac1 floxed + STZ + gly vs. Epac1 floxed + STZ, and ^$^ *p* < 0.05 is Epac1 CreLox + STZ + Gly vs. Epac1 CreLox + STZ. *n* = 5 for all groups.

Increased permeability is an early event in diabetic retinopathy [22,23]. In these studies, we used the Epac1 floxed and Epac1 CreLox mice with diabetes only or treated with glycyrrhizin for two and six months to investigate the role of Epac1 and glycyrrhizin on permeability in the diabetic retina. Figure 1 shows that diabetes significantly increased permeability using both fluorescein angiograms and Evan’s blue techniques at both two (top) and six months (bottom). Glycyrrhizin reduced the diabetes-induced permeability in both the Epac1 floxed and Epac1 CreLox mice. Glycyrrhizin had no effects on permeability in mice that were not diabetic.

### 3.2. Glycyrrhizin Increased Retinal Thickness and Cell Numbers in the Ganglion Cell Layer of 2 Month Epac1 Floxed and Epac1 CreLox Diabetic Mice

**Figure 2:**
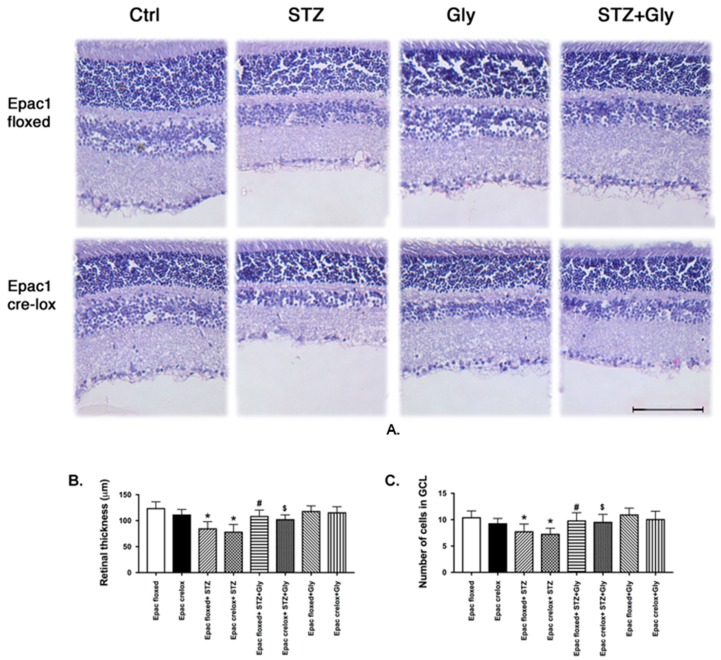
Representative images of the retina, (**A**) Neuronal measurements of retinal thickness (**B**), and cell numbers in the ganglion cell layer (GCL, (**C**)) in Epac1 floxed and Epac1 CreLox mice alone or treated with glycyrrhizin. Data are mean ± SEM * *p* < 0.05 vs. Epac1 floxed, # *p* < 0.05 is Epac1 floxed + STZ + gly vs. Epac1 floxed + STZ, and $ *p* < 0.05 is Epac1 CreLox + STZ + Gly vs. Epac1 CreLox + STZ. *n* = 5 for all groups.

We have previously reported that Compound 49b, a β-adrenergic receptor agonist, increased retinal thickness and cell numbers in the ganglion cell layer in mice [18]. Since Epac1 can signal downstream of β-adrenergic receptors, we made the Epac1 floxed and Epac1 CreLox mice diabetic only or treated them with glycyrrhizin, a HMGB1 inhibitor, for these studies. We have previously shown that Epac1 can inhibit HMGB1 [24]. Figure 2 demonstrates that diabetes causes retinal thinning and cell loss in the ganglion cell layer of both Epac1 floxed and Epac1 CreLox mice. Glycyrrhizin maintained normal retinal thickness and cell numbers in both groups of mice at two months of age. Glycyrrhizin alone (without diabetes) had no effect on neuronal changes.

### 3.3. Glycyrrhizin Reduced Degenerate Capillary Numbers in Both Epac1 floxed and Epac1 CreLox Diabetic Mice

**Figure 3:**
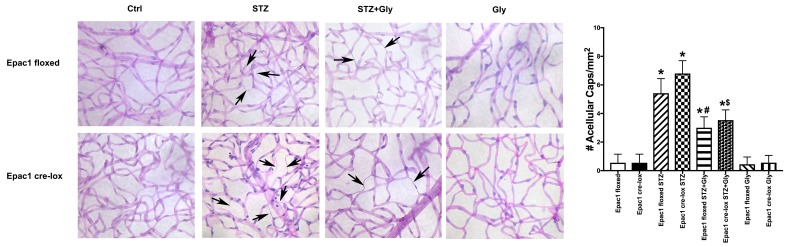
Degenerate capillaries in Epac1 floxed and Epac1 CreLox mice alone or treated with glycyrrhizin. Data are mean ± SEM * *p* < 0.05 vs. Epac1 floxed, # *p* < 0.05 is Epac1 floxed + STZ + gly vs. Epac1 floxed + STZ, and $ *p* < 0.05 is Epac1 CreLox + STZ + Gly vs. Epac1 CreLox + STZ. *n* = 5 for all groups.

Figure 3 shows that diabetes significantly increased degenerate capillary numbers in both the Epac1 floxed and Epac1 CreLox mice, as we have reported in other mice [18]. Glycyrrhizin in the drinking water significantly reduced degenerate capillary numbers in both strains of mice, suggesting that HMGB1 inhibition is effective in protective the retinal vasculature, despite the presence or absence of Epac1. Glycyrrhizin had no effect on the mice without diabetes induction.

### 3.4. Epac1 and Glycyrrhizin Work Synergistically to Reduce Reactive Oxygen Species (ROS) at 6 Months of Diabetes

**Figure 4:**
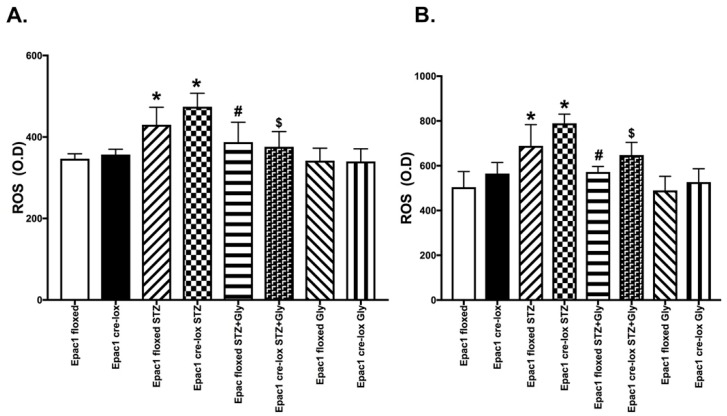
Reactive oxygen species (ROS) measurements at 2 months (**A**) and 6 months (**B**) of diabetes (STZ) in Epac1 floxed and CreLox mice alone or treated with glycyrrhizin. Data are mean ± SEM. * *p* < 0.05 vs. Epac1 floxed, # *p* < 0.05 is Epac1 floxed + STZ + gly vs. Epac1 floxed + STZ, and $ *p* < 0.05 is Epac1 CreLox + STZ + Gly vs. Epac1 CreLox + STZ. *n* = 5 for all groups.

Diabetic retinopathy is associated with increased oxidative stress [25]. Figure 4 shows similar reductions in ROS by glycyrrhizin in both Epac1 floxed and Epac1 CreLox mice at both two (Figure 4A) and six months (Figure 4B). Interestingly, at 6 months of diabetes, Epac1 and glycyrrhizin displayed the trend to work synergistically to reduce ROS, as the levels in Epac1 floxed + STZ + glycyrrhizin were reduced more than Epac1 CreLox + STZ + glycyrrhizin. Glycyrrhizin alone had limited effects on ROS without diabetes, and Epac1 levels did not appear to reduce ROS without the stressor of diabetes.

### 3.5. Glycyrrhizin Reduced TNFα and IL-1β Levels at Both 2 and 6 Months in Epac1 Mice

**Figure 5:**
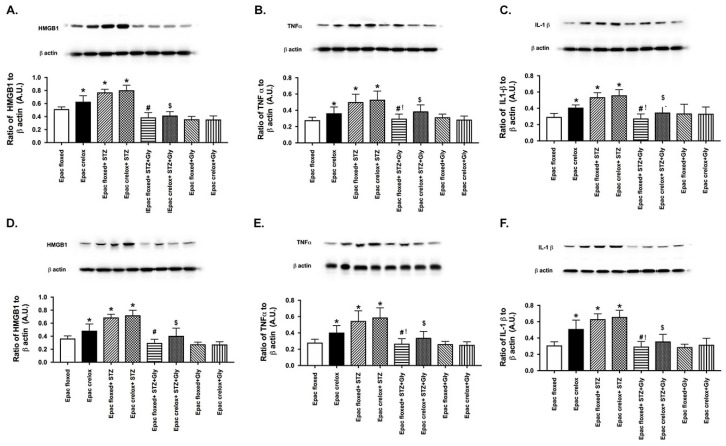
HMGB1 (**A**,**D**), TNFα (**B**,**E**) and IL-1β levels (**C**,**F**) at 2 months (**top**) and 6 months (**bottom**) of diabetes (STZ) in Epac1 floxed and CreLox mice alone or treated with glycyrrhizin. Data are mean ± SEM. * *p* < 0.05 vs. Epac1 floxed, # *p* < 0.05 is Epac1 floxed + STZ + gly vs. Epac1 floxed + STZ, $ *p* < 0.05 is Epac1 CreLox + STZ + Gly vs. Epac1 CreLox + STZ, and ^!^ *p* < 0.05 is Epac1 CreLox + STZ + gly vs. Epac1 floxed + STZ + gly. *n* = 5 for all groups.

We have previously shown that diabetes increased HMGB1 levels, which was inhibited by glycyrrhizin [14]. We wanted to determine if Epac1 had any effect on the glycyrrhizin-induced actions on TNFα and IL-1β. Figure 5 shows that diabetes increased TNFα and IL-1β in both Epac1 floxed and Epac1 CreLox mice. Glycyrrhizin reduced both levels of both proteins in each set of mice, with a stronger response in the Epac1 floxed mice vs. the Epac1 CreLox mice. This suggests that Epac1 and glycyrrhizin may work synergistically in regulating anti-inflammatory actions.

### 3.6. Epac1 and Glycyrrhizin Regulated SIRT1 in the Epac1 Floxed Diabetic Mice

**Figure 6:**
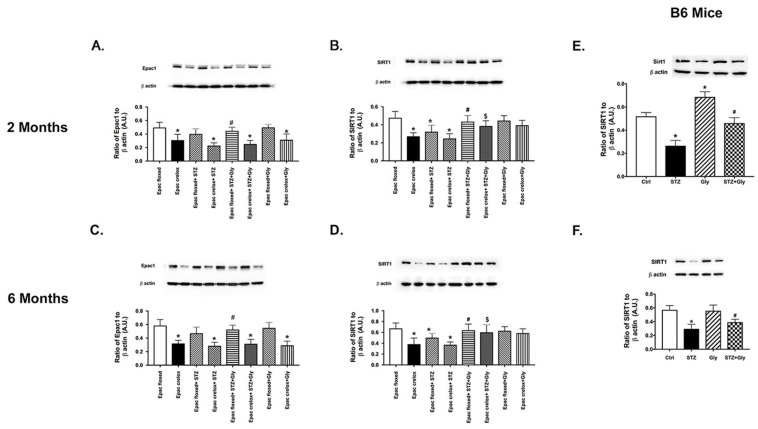
Epac1 (**A**,**C**) and SIRT1 (**B**,**D**) in diabetic Epac1 floxed and Epac1 CreLox mice alone or treated with glycyrrhizin. (**E**,**F**) are SIRT1 levels in diabetic B6 mice treated with glycyrrhizin. Data are mean ± SEM. * *p* < 0.05 vs. Epac1 floxed, # *p* < 0.05 is Epac1 floxed + STZ + gly vs. Epac1 floxed + STZ, $ *p* < 0.05 is Epac1 CreLox + STZ + Gly vs. Epac CreLox + STZ. For the B6 mice, * *p* < 0.05 vs. ctrl, and # *p* < 0.05 vs. STZ alone. *n* = 5 for all groups.

Figure 6 demonstrated that diabetes reduced SIRT1 levels in both groups of mice, with a stronger response in the Epac1 CreLox mice, suggesting that Epac1 regulates SIRT1. Interestingly, glycyrrhizin significantly increased SIRT1 in both Epac1 floxed and CreLox mice. Since SIRT1 can regulate HMGB1 [26], this may offer an additional mechanism by which glycyrrhizin can regulate HMGB1 actions. Due to the novelty of this finding, we also tested SIRT1 levels in C57Bl/6J mice treated with glycyrrhizin for 2 and 6 months. We found that diabetes reduced SIRT1 at both time points, but that glycyrrhizin significantly increased levels at 2 and 6 months (Figure 6E,F).

## 4. Discussion

Diabetes has been increasingly accepted as an inflammatory disease [1,2], with a great deal of recent work on the inhibition of this inflammation. More recently, research has suggested that chronic hyperglycemia may represent a form of a sterile inflammation and activate DAMPs. We recently reported that glycyrrhizin inhibited HMGB1 in the diabetic retina through anti-inflammatory mechanism [15]. Our findings agreed with more acute studies in diabetic rats [13]. Since we have previously shown that Epac1 reduced HMGB1 in REC in vitro [7,10], we wanted to investigate whether Epac1 worked upstream of HMGB1 to protect the diabetic retina.

Using Epac1 endothelial cell specific knockout mice, we found that diabetic Epac1 floxed mice had similar permeability, neuronal, and vascular damage compared to diabetic Epac1 CreLox mice. These findings were surprising based upon other studies showing that Epac1 reduced permeability changes [9] and inflammatory mediators [7,27]. We expected Epac1 alone to be protective the diabetic retina. Our data does confirm cell culture studies showing that Epac1 reduced HMGB1 actions in the retinal vasculature [24,28].

We further expanded these findings by treating the diabetic Epac1 floxed and diabetic Epac1 CreLox mice with glycyrrhizin to investigate whether Epac1 and glycyrrhizin would work synergistically in the retina. There is a great deal of literature suggesting that glycyrrhizin or glycyrrhizic acid is anti-inflammatory through inhibition of HMGB1 [11,14,29,30]. Interestingly, Epac1 and glycyrrhizin worked synergistically to reduce TNFα and IL-1β levels in the retinal lysates, suggesting that both pathways are anti-inflammatory in the diabetic retina. While Epac1 and glycyrrhizin were synergistic in their actions on the inflammatory mediators, glycyrrhizin alone was enough to significantly reduce permeability, neuronal, and vascular damage in both the Epac1 floxed and Epac1 CreLox mice. The presence or absence of Epac1 did not appear to alter these measurements in the mice.

In addition to finding that Epac1 and glycyrrhizin can both protect the retina against diabetes-induced damage, we also unexpectedly observed that glycyrrhizin reduced SIRT1 levels in the retina of Epac1 mice, as well as in C57BL/6 mice. We chose to investigate SIRT1 levels as a number of studies have shown that SIRT1 can deacetylate HMGB1 to reduce its cytoplasmic levels [26]. SIRT1 is key to reduced NFkB, COX2, and other deleterious pathways involved in diabetic retinopathy [31]. Work in the diabetic kidney had suggested that glycyrrhizic acid could regulate HMGB1 through activation of SIRT1 in both cell culture and db/db mice [32,33]. Our findings may represent the first time showing that glycyrrhizin increased SIRT1 in the diabetic retina.

This study was done using glycyrrhizin as a preventative treatment. Future studies will be done to determine if Epac1 and glycyrrhizin also work together when glycyrrhizin treatment is initiated after diabetic complications have occurred. Additionally, Epac1 was only eliminated in endothelial cells. It is likely that glycyrrhizin affects multiple retinal cell types, while Epac1 actions were only investigated in the retinal vasculature. Future work may use whole animal knockout studies or other Cre lines to test specific retinal cell types.

## 5. Conclusions

In conclusion, we found that Epac1 is protective the diabetic retina, which agrees with our prior studies in cell culture and the ischemia/reperfusion model. We also found that Epac1 and glycyrrhizin work together to reduce inflammatory mediators in the diabetic retina. Glycyrrhizin increased SIRT1 levels in the diabetic retina, which may offer a novel mechanism by which glycyrrhizin inhibits HMGB1 actions. Finally, both Epac1 and glycyrrhizin prevented diabetes-induced permeability, neuronal and vascular damage to the retina.

## Figures and Tables

**Table 1:** Data are mean ± standard deviation (SD).

	Epac Floxed								Epac1 Cre-Lox							
	Epac +/+		Epac +/+ +STZ		Epac +/+ STZ + Gly		Epac +/+ Gly		Epac ^−/−^		Epac ^−/−^ +STZ		Epac ^−/−^ STZ + Gly		Epac ^−/−^ Gly	
	BW (g)	BG (mg/dL)	BW (g)	BG (mg/dL)	BW (g)	BG (mg/dL)	BW (g)	BG (mg/dL)	BW (g)	BG (mg/dL)	BW (g)	BG (mg/dL)	BW (g)	BG (mg/dL)	BW (g)	BG (mg/dL)
3m	24.6 ± 2.2	103 ± 15	25.1 ± 2.1	109 ± 6.7	25.2 ± 2.0	112 ± 9.1	25.3 ± 1.8	108 ± 7.2	24.6 ± 2.2	110 ± 11	25.8 ± 1.9	107 ± 6.9	25.5 ± 2.0	104 ± 8.8	25.3 ± 2.8	103 ± 7.9
3m + 2m STZ	26.5 ± 1.7	112 ± 10	22.1 ± 4.9 *	463 ± 60 ^#^	24.9 ± 1.7 *	448 ± 57 ^#^	26.3 ± 1.4	115 ± 13	28.3 ± 2.0	126 ± 26	23.5 ± 2.1 *	447 ± 77 ^#^	24.4 ± 1.3 *	419 ± 69 ^#^	26.6 ± 2.3	114 ± 18
3m + 6m STZ	38.2 ± 4.5	112 ± 12	31.6 ± 2.1 *	411 ± 74 ^#^	32 ± 2.0 *	416 ± 76 ^#^	36.3 ± 2.9	124 ± 15	38 ± 3.1	114 ± 12	30.8 ± 3.2 *	438 ± 79 ^#^	31.5 ± 2.3 *	422 ± 73 ^#^	36 ± 1.8	124 ± 23

* *p* < 0.05 vs. ctrl for BW ^#^ *p* < 0.05 vs. ctrl for blood glucose (BG) in mg/dL; body weight is expressed in grams (g). Three months are controls; 3m + 2m STZ are 2 months diabetes if treated with STZ; 3m + 6m STZ are 6 months of diabetes if treated with STZ. STZ, streptozotocin; Gly, glycyrrhizin.
